# Commentary: Lactobacilli Dominance and Vaginal pH: Why Is the Human Vaginal Microbiome Unique?

**DOI:** 10.3389/fmicb.2017.01815

**Published:** 2017-09-25

**Authors:** Virginia Fuochi, Giovanni Li Volti, Pio Maria Furneri

**Affiliations:** ^1^Microbiology Section, Department of Biomedical and Biotechnological Sciences, University of Catania Catania, Italy; ^2^Medical Biochemistry Section, Department of Biomedical and Biotechnological Sciences, Biological Tower, University of Catania Catania, Italy

**Keywords:** *Lactobacillus* spp., vaginal microbiome, pH, estrogens, lactic Acid

Vaginal microbiota is involved in the homeostasis of mammal female's physiological conditions (Jašarević et al., 2015), avoiding vaginal infections such as aerobic vaginitis, bacterial vaginosis, and candidiasis (Vitali et al., 2007; Tempera and Furneri, 2010; Heczko et al., 2015). To this regard, the *genus Lactobacillus* plays a major role in women vagina, exerting its beneficial functions by lactic acid and bacteriocins production (Fuochi et al., 2017; Tachedjian et al., 2017). Furthermore, the very low pH (4.5–6.0) of the genital tract should be taken into due account in protection of the host (Wylie and Henderson, 1969). Miller et al. have analyzed and put together possible hypotheses (Figure 1) in order to explain the important correlation between Lactobacilli dominance and vaginal pH in human species. They further elaborated their own theory as a possible explanation of that peculiarity. In particular, the authors suggested that the high level of glycogen, present in the human females' vagina but not in other mammal species, could be an energy source for *Lactobacillus* growth. Such presence should be due to the human diet particularly rich in starch. Although, we do share Miller's exhaustive hypotheses and conclusions, we would like to point out additional aspects that have to be taken into due account. On the one hand, vaginal epithelium is rich in glycogen as well as cervico-vaginal mucus is rich in mucins and glycoproteins and therefore there are very high levels of nutrients allowing colonization and dominance of lactobacilli (Martín Rosique et al., 2008; Mirmonsef et al., 2014; Nunn and Forney, 2016). On the other hand, high glycogen concentrations are dependent on the high levels of estrogen (Mirmonsef et al., 2016). Moreover, Gorodeski et al. (2005) provided a further explanation for vaginal pH: vaginal cells seem to contribute to luminal acidity through an active and estrogen-dependent proton secretion throughout the woman's life.

**Figure 1:**
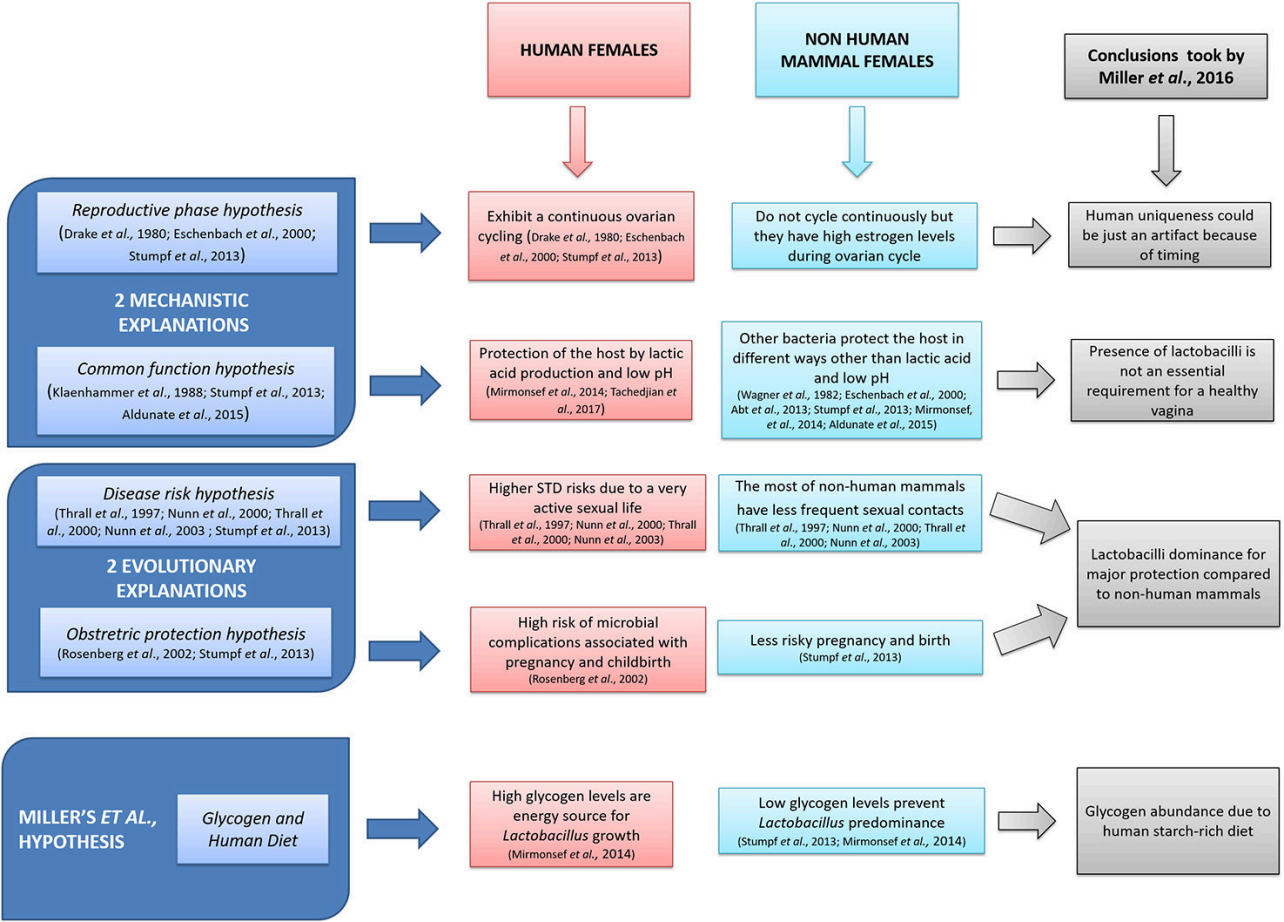
Miller et al. (2016): a schematic representation.

Furthermore, women's life is characterized by continuous physiological changes, from their birth through the reproductive age to menopause, and during all these phases the vaginal microbiota and vaginal epithelium radically change (Paavonen, 1983). The fertile age is characterized by monthly ovulation and therefore high estrogenic levels which, also thanks to the action of proton pumps (Aldunate et al., 2015; Mirmonsef et al., 2016), lead the vaginal lumen to be particularly acid. The combined effect of the presence of glycogen and low pH render provide the best conditions for the growth of *Lactobacillus*.

When the woman reaches the menopause this pattern changes significantly because estrogens are no longer present and the glycogen level decreases leading to a significant modification of vaginal microbiota characterized in particular by a decrease in the amount of lactobacilli (Roccasalva et al., 2002; Hickey et al., 2012; Nunn and Forney, 2016; Shen et al., 2016). The importance of estrogens is further substantiated by the evidence that restoring estrogen levels results in improved trophic conditions of the vaginal epithelium restoring all the benefits arising from such condition (Paavonen, 1983; Roccasalva et al., 2002; Gorodeski et al., 2005; Lien et al., 2009; Hickey et al., 2012; Nunn and Forney, 2016; Shen et al., 2016).

As far as the diet theory is concerned, different vaginal ecotypes should exist according to the different types of diets in the same way as different habits and practices (Lien et al., 2009; Hickey et al., 2012), and this would be accentuated in cases of intolerance or allergies. In particular, in patients with celiac disease, women might have a very different vaginal microbiota, and therefore it would be very interesting to carry out a study to confirm a closer relationship between diets and vaginal microbiota.

In conclusion, we believe that Miller's observations are of great interest for the field, but in our opinion the explanation is more related to the mechanistic theories (reproductive phase hypothesis and common function hypothesis) than it is to the others. Nevertheless, we believe that uniqueness of human vaginal microbiome is due to all five theories (including Miller's hypothesis), intersecting each other, and from which it is impossible to extrapolate one single mechanistic theory.

## Author contributions

All authors listed have made a substantial, direct and intellectual contribution to the work, and approved it for publication.

### Conflict of interest statement

The authors declare that the research was conducted in the absence of any commercial or financial relationships that could be construed as a potential conflict of interest.
